# The Anterior Cruciate Ligament: Principles of Treatment

**DOI:** 10.7759/cureus.40269

**Published:** 2023-06-11

**Authors:** Prannoy Shom, Anuj R Varma, Roshan Prasad

**Affiliations:** 1 Orthopaedics, Jawaharlal Nehru Medical College, Datta Meghe Institute of Higher Education and Research, Wardha, IND; 2 Medicine, Jawaharlal Nehru Medical College, Datta Meghe Institute of Higher Education and Research, Wardha, IND; 3 Medicine and Surgery, Jawaharlal Nehru Medical College, Datta Meghe Institute of Higher Education and Research, Wardha, IND

**Keywords:** anterior cruciate ligament (acl), principles of treatment, knee joint, acl repair, acl reconstruction

## Abstract

The anterior cruciate ligament (ACL) is a crucial connective tissue in the knee joint (tibiofemoral joint). Although the surgical anatomy of this ligament has been studied and interpreted for decades, it remains a topic of discussion among surgeons. The ACL has two bundles, the anteromedial (AM) and the posterolateral (PL) bundle. ACL tears are among the most frequently sustained injuries to the tibiofemoral joint. The ACL is an important rotational stabilizer of the knee joint. The human knee joint can be classified as a complex structure, as it has many ligaments supporting its stability and ensuring required joint mobility. Previously, the outcomes of primary ACL surgery were poor; however, with time, the modalities have improved substantially. There are two methods of performing the reconstruction procedure, the single-bundle method, in which only the AM bundle is reconstructed, and the double-bundle method, in which both the AM and PL bundles of the ACL are reconstructed. Double bundle arthroscopic ACL reconstruction has been recognized as the gold standard procedure. The grafts used for the reconstruction procedure are the tendon of the patella graft and the grafts of the hamstrings. However, one of the drawbacks of performing this surgery is the development of complications, like osteoarthritis. This complication is observed majorly in sports professionals. This article aims to sum up the anatomy of the ACL, its regular tears, the various surgical aspects of managing it, and the advancement of treatment options in the past centuries. Although much has been achieved, detailed scientific studies should be carried out to improve the prognosis and decrease the risk of development of complications.

## Introduction and background

The knee joint is a hinge joint formed by articulating two bones, the femur and the tibia. The stability of this joint rests upon its ligaments. There are a total of 11 ligaments that stabilize the knee joint, among which the anterior cruciate ligament (ACL) is crucial for preventing translocation of the tibia over the femur. The knee joint is subjected to many forces in our day-to-day lives, with sports proving to be a significant contributory factor. The ACL is the most frequently ruptured ligament in the human body. It is often associated with the tearing of the fibers of the medial and lateral collateral ligaments, owing to their proximity to the ACL [1].

The ligament may get avulsed from any one of its attachments. The severity may depend on the mechanism of injury ranging from a sprain to a complete tear of the ACL. There may even be an avulsion of a small piece of bone accompanying the ligament, which can be visualized on the X-rays. It can occur as an isolated ACL tear or involvement of a multi-ligamentous injury, depending on the mechanism and severity of the damage. Another rare type of injury is knee dislocation leading to multi-ligamentous damage. Clinical features of complete ACL tear include pain, swelling secondary to hemarthrosis, and instability in case of chronic tears. Clinically ACL tears can be diagnosed by the anterior drawer test and the Lachman test [1].

After proper diagnosis with the help of clinical examination, radiological investigations and arthroscopic examinations, management is decided based on the severity of the injury. Conventionally ACL tears were managed conservatively, especially grade I and grade II tears. With the advent of newer surgical techniques and arthroscopy, treating ACL tears has gained much importance. Once the swelling subsides, physiotherapy is beneficial in regaining strength. However, with the advancement of medical science, operative procedures have been proven to have better prognoses and are now given preference when choosing treatment options. Surgery is the first line of treatment in case of an injury sustained by the athletes [1].

Management of ACL tears includes acute repair of avulsion fractures from femoral or tibial attachment and reconstruction for chronic tears. The graft used for reconstruction can be autograft (from the same person), allograft (from a different person, same species), or synthetic graft. Different grafts used are bone-patellar tendon grafts, hamstring tendons (semitendinosus and gracilis), and perineal tendons in case of multi-ligamentous reconstructions. Fixation of grafts can be done with the help of screws, with the most recent advent being bio-absorbable screws [1].

## Review

Methodology

**Figure 1:**
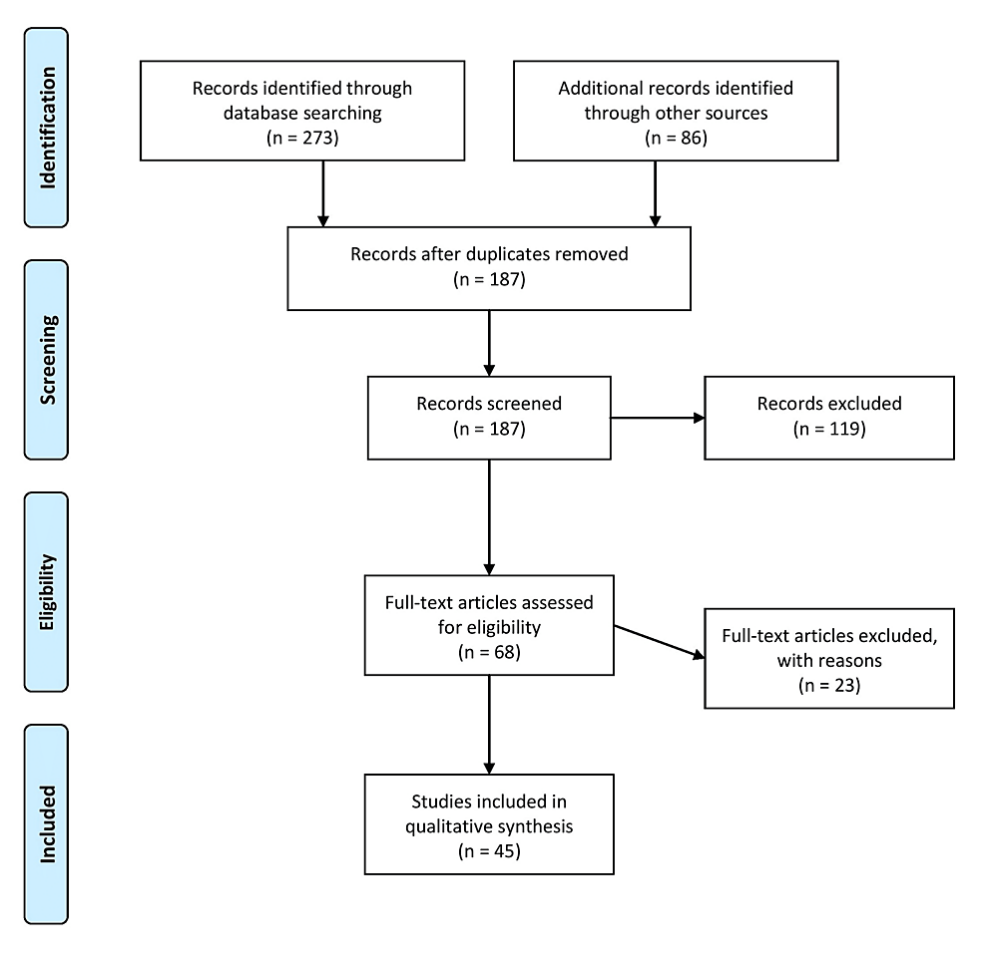
The selection process of articles used in this study Adopted from the Preferred Reporting Items for Systematic Reviews and Meta-Analyses (PRISMA).

A comprehensive literature search was conducted using electronic databases such as PubMed, Medical Literature Analysis and Retrieval System Online (MEDLINE), and the Cochrane Library. The search encompassed articles published from the year 2000 to the present. It utilized specific keywords such as "anterior cruciate ligament (acl)," "principles of treatment," "knee joint," "acl repair," and "acl reconstruction." The articles were screened for relevance and eligibility based on inclusion and exclusion criteria. The inclusion criteria required that the articles be published in English; report on ACL tear and treatment used, associated factors and rehabilitation achieved; report on both observational and interventional studies published and not be duplicates. The exclusion criteria required that the articles be published in non-peer-reviewed journals. Figure 1 describes the selection process of articles used in our study.

Anatomy and function of the ACL

The ACL consists of two bundles, the anteromedial (AM) and the posterolateral (PL) ligament. These bundles fan out along the medial wall of the lateral femoral condyle and insert into the tibial plateau. They are separated by a connective tissue septum, which contains the vascular supply. The septum also ensures synergic movement of the bundles, maintaining the full range of motion [2]. The AM bundle remains taut throughout the knee flexion from 45 to 60 degrees. The PL bundle is taut in extension and relaxes with the flexion of the tibiofemoral joint. This mechanism allows the knee to be rotated. Thus, the bundles of the ACL, the AM and the PL, are responsible for anteroposterior stability and rotatory stability, respectively [3]. The lateral bifurcate ridge and the lateral intercondylar ridge are important anatomical landmarks for the placement of tunnels during the reconstruction procedures. The bifurcate ridge is posterior to the lateral intercondylar ridge, an insertion location for the AM, besides PL bundles of ACL. The lateral bifurcate ridge is felt and approximated in about 75% of patients [4-6].

Available methods to treat ACL injury: conservative vs surgical

Athletes undergo immense pressure during their recovery period. Resting and rehabilitation are tough for these active individuals, and the scrutiny associated with their comeback performance becomes patronizing. Therefore, receiving the best possible intervention is paramount to them [7-11]. Conventional methods still need to provide desirable recovery. Hence, ACL reconstruction becomes the best surgical intervention for these patients. It ensures faster recovery and helps them regain their form [10-12].

The frequency of meniscal injuries in patients with ACL deficiency has higher incidence rates in skeletally undeveloped patients. Meniscal tears do not delay ACL reconstruction surgery. In fact, meniscal treatment and ACL reconstruction are done in a single sitting. It signifies that a postponement in handling ACL injuries could be linked etiologically to these meniscal tears. As a result, deciding whether to go or not for surgical intervention among these patients must be considered [13].

ACL repair (conservative procedure)

History

The ancient Greeks first described ACL injuries. Mayo Robson was a pioneer in performing ACL repair surgery in the era of late 1800s [14]. He demonstrated the management of ACL tears by repairing femoral attachment sites by the end of the 19th century. By the end of the 19th century, ACL repair was improved, becoming the treatment of choice for most surgical procedures [15-17]. Despite the initial results of ACL repair being promising, the follow-up of the patients presented with significant issues, which could be addressed. It was found that the ACL re-ruptured at the rate of about >50% at five years of interval post-operatively [15,16]. Around the same time, a new procedure of ACL reconstruction surgery was being developed, and many randomized clinical trials were conducted. These trials showed improvement in the patients post-operatively compared to the primary ACL repair technique [18,19]. Slowly, ACL reconstruction surgery replaced ACL repair and became the standard management procedure.

Van der List et al. described various factors that led to a shift in the primary choice of treatment from repair to reconstruction. The primary among them was ACL repair, an open method that resulted in subsequent morbidity due to arthrotomy. The arthroscopic technique became advanced only in the late 1990s and early 2000s. This has been accompanied by novel rehabilitation techniques, leading to early mobilization post-operatively [20].

Finally, the most important factor leading to a shift from ACL repair to ACL reconstruction was that the repair technique did not consider the tear's location in its procedure. Sherman et al. recognized that the tear's site substantially influenced the ACL repair results [21]. All these factors have led to the total abandonment of the ACL repair procedure in favor of a new advanced technique of ACL reconstruction. Since the past three years, there has remained a substantial rise in interest in primary ACL repair, and this has subsequently led to newer studies being carried out regarding the same [22].

New Techniques of ACL Repair

Although reconstruction remains the gold standard procedure, ACL repair can still be beneficial in managing ACL injuries. Acute avulsions from the tibial or femoral attachment are amenable to repairs. Novel approaches have improved rapidly in the past few years and are equally efficient compared to newly developed arthroscopic ACL reconstruction. Better investigating modalities like magnetic resonance imaging (MRI) help localize and visualize the tear more accurately [23-25]. However, the primary ACL repair procedure has scope for improvement and requires modifications [22].

ACL reconstruction (surgical procedure)

History

From the initial years of the 20th century, piqued interest in ACL repair led to the proposal of utilizing sutures as a treatment modality. However, only during the 1970s could an accurate diagnosis of ACL injury be made by examining clinically. The anterior drawer test, which involves knee flexion at 90 degrees, is acceptable. The drawback of the anterior drawer test is that it cannot be done in acute tears, where the Lachman test is useful. The pivot shift test, as described by Galway, coupled with the Lachman test, introduced by Torg, proved to be a major milestone in diagnosing ACL tears and made up for the insufficiencies of previous diagnostic modalities. These tests were useful to the patient and the clinician as they could point to when the patient sustained their injury. It also played an important role in understanding the dynamics of the ACL ligament [26-29].

The early 2000s ushered in the usage of patellar tendon grafts, with Franke being a pioneer in the introduction of the same. His presentation in Lyon, at the first International Society of Knee meeting in 1978, involved a re-evaluation of his 1976 article. He proposed exploitation of the mid-one-third of the patellar tendon as a free graft, henceforth affording its faultless structural location. This operation became very popular, soon being touted as the gold standard treatment for ACL grafts. After much utilization as a treatment modality, patellar tendon graft soon found its disadvantages, including the danger of patellar fracture and minor complications like patellar tendinitis and anterior knee pain [30,31]. A "hamstring graft" was proposed as a solution to the problems the patellar tendon grafts faced. The first description of this procedure is attributed to Cho et al., who altogether used the grafts of semitendinosus and gracilis to rebuild the ACL [32]. These two choices, the graft of the tendon of the patella and the hamstring graft, are widespread today through minimal alterations in various fixation procedures [26].

Current Scenario

There have been several debates around the globe regarding the principles of the ACL reconstruction procedure: precisely, the schedule of reconstruction and rehabilitation and the kind of graft to be used [33]. Although, it would be correct to say that the double-bundle technique is more efficient than the single-bundle technique [34].

Repair and Rehabilitation

**Figure 2:**
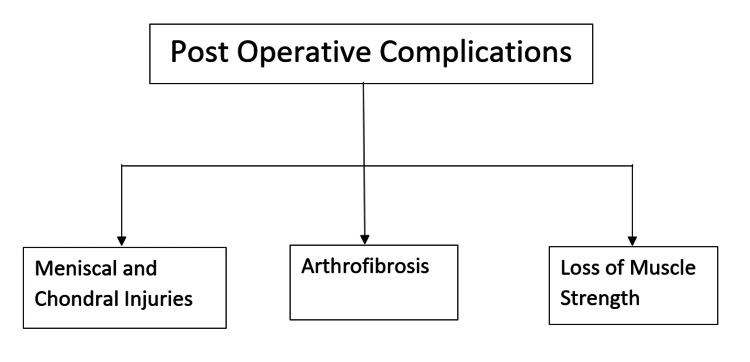
Post-operative complications The author re-created from the source [35].

Three key aspects must be considered while performing the ACL reconstruction procedure. First, there is an increased danger of meniscal and chondral injuries after delayed ACL reconstruction. In addition, there are chances of developing arthrofibrosis due to premature ACL reconstruction. Finally, the patient might also lose muscle strength and wasting due to delayed reconstruction [35]. The number of patients suffering a primary meniscal tear and an ACL tear accounts for about 11% to 50% [36]. The chances of developing chondral lesions like cartilage deterioration and cartilage degeneration increase significantly after an ACL tear. About 20% of ACL tears may develop cartilage deterioration, whereas 30% may suffer from cartilage degeneration [37]. Figure 2 shows the post-operative complications.

Arthrofibrosis can be defined as the stiffness of the joint that averts the retainment of the complete range of motion. It is commonly seen in premature ACL reconstruction. According to Shelbourne et al., about 50% of the patients who had their operation within one week of developing injury had lost 5˚ extension. On the other hand, only 17% of the patients developed arthrofibrosis and underwent surgical intervention from the second week to the third week [38]. Although, Wasilewski et al. noted a loss of extension in patients who underwent ACL reconstruction after six months of injury [39]. Preoperative therapy programs are likewise vital to regain muscle strength and prevent wasting of the muscles until surgery is performed. A quadriceps strength deficit of about 20% was noted among the vitally deprived experimental outcomes [40].

Types of Graft

For ACL reconstruction procedures, the hamstrings and patella tendons are frequently used. Both are efficient and ensure excellent prognosis [41]. Allografts used for ACL reconstruction include the tendon of the patella, Achilles tendon, and tendon of the tibialis. In comparison, autografts could be the tendons of the hamstrings and patella. Autografts are more frequently used. However, both are equally efficient and ensure an excellent prognosis. Table 1 shows the differences between autograft and allograft.

**Table 1:** Autograft vs allograft The author self-created the table.

Autograft	Allograft
It is a graft that is taken from the same individual in whom the surgery is being performed	The graft used in this is taken from a human cadaver
Examples: hamstring tendons and patellar tendon	Examples: Achilles tendons and patellar tendon

Single- and Double-Bundle Reconstruction Surgery

Abundant research has established the scientific and biomechanical aids of single- and double-bundle surgery [42]. Some research groups have shown that post-operatively, double-bundle procedures have superior knee stability [43]. In contrast, other study groups have shown that there is very minimal to no difference in the strength of the knee after any of the above procedures were performed [44]. Even though the double-bundle reconstruction surgery is technically demanding, this procedure well reinstates the anatomical biomechanics of the knee. It delivers improved rotational stability compared to a single-bundle reconstruction surgery [45].

Discussion

The ACL is among the frequently injured ligaments of the knee joint. It has namely three grades of tear. Tear grades I and II can be managed conservatively by immobilization techniques. On the other hand, grade III tears need surgical intervention. They must be handled by primary ACL repair, which is only indicated in avulsion fractures of ACL or ACL reconstruction technique with the help of grafts, namely the tendon of the patella and hamstrings. Based on the anatomical location of the two bundles of ACL (the AM and PL), the reconstruction procedure can be a single or double bundle. The ACL reconstruction procedure is currently the first line of treatment, with the double-bundle reconstruction technique the preferred treatment modality, as it provides better stability and maintains the proper anatomical biomechanics of the tibiofemoral joint.

## Conclusions

The tibiofemoral hinge joint is among the most flexible joints in the body. The ACL has two bundles, the AM and the PL bundle, which are named per their anatomical position. The ACL is one of the most frequently injured ligaments of the inferior extremity, especially in young sportspersons, necessitating prompt and adequate medical and surgical care. Conservative procedures, the initial treatment of choice, have a major drawback of late rehabilitation and greater chances of re-rupturing the ACL. Surgical procedures are the mainstay of management and consist of two procedures, primary ACL repair, which is the older method of management, and the newly developed arthroscopic ACL reconstruction, namely single-bundle and double-bundle type. ACL reconstruction is considered the surgical technique of choice owing to its relatively low rate of complications and early recovery. Despite being the gold standard treatment today, it brings forth the drawbacks of arthrofibrosis, which, though rare in occurrence, can prove to be troublesome if not treated on time.
